# St. George's Hospital

**Published:** 1900-01-13

**Authors:** 


### ST. GEORGE'S HOSPITAL.

Mr. Timothy Holmes writes the following letter to the *Times* :—

"The annual distribution of the Prince of Wales's Hospital Fund has recently been made, and the particulars were announced in your issue for December 22nd. Of all the great metropolitan hospitals which applied for a share in that distribution, St. George's was the only one refused. This refusal was accompanied by the remark that its cause was the pecuniary position of the hospital, while free praise was given to its management and to the efforts on improving its internal arrangements, on which a sum considerably exceeding £20,000 is now being expended. Now this must tend to confirm an opinion, which we hear is widely entertained, that St. George's Hospital is too rich to need support; and we would ask your aid in removing such an impression, which is wholly unfounded. True it is that St. George's is not in so precarious a position as many of our metropolitan hospitals are; but the following rapid summary of its finance will enable your readers to see that it is in very real need of help. The year 1898 is, of course, the last for which complete accounts are ready, but those for 1899 tell much the same general tale. The income is made up of two main items—ordinary £25,197, and extraordinary £11,844. The expenditure divided in the same way shows ordinary £34,452, and extraordinary £8,766. Thus the ordinary expenditure shows an excess of £9,255 over the ordinary income. And the ordinary expenditure is constantly rising as new and more efficient methods of treatment are devised, as it becomes necessary to spend more on nursing, and as the comfort of the nurses and servants is better provided for. The statistical table published in the annual report shows that for the decennium 1873 to 1883 the average expenditure was not much over

£23,000, and for the next decade under £26,000, since which it has been steadily rising, and may now be taken at £35,000. It ought also to be known that, in consequence of its possessing a country branch at Wimbledon (not a mere convalescent home) to which the less acute cases are sent, St. George's admits probably a greater proportion of serious illness than almost any other hospital. The ordinary income is made up chiefly of (1) subscriptions and donations; (2) dividends on realised property and rents. It is in the first-named of these sources of income that the Governors have to lament a great falling-off. The average of subscriptions and donations from 1873 to 1883 was over £10,600, and in the next decade over £9,200. It is now below £8,000, and that in spite of continuous appeals and efforts to obtain new subscribers, and in spite of the wealth of the district in which thee hospital stands. And there can be no doubt that the cause of the limited subscription is the general belief that the hospital has immense realised property and is in no want—a belief which the governors fear the report of the Prince of Wales's Fund will tend to confirm. It is true that St. George's has received in the last 30 years several large legacies, so that the amount received for dividends has risen from an average of under £4,000 in the decade first named to £13,662 in the year 1898. The rents remain at about the same sum (£1,600) during the last few years. But it will be seen at a glance that the increase in realised income does not balance the increase in ordinary expenditure—the former being under £10,000 and the latter about £12,000. In other words, the hospital is not now in so good a pecuniary position as in the period between 1873 and 1883, except (for the Governors have no wish to overstate their case in any way) that it has a larger fund to fall back upon as it becomes necessary to sell out stock. The Governors have always freely acted upon the conviction that the money bequeathed to the hospital by testators is not given to them to hoard up, and so to save the present managers the trouble of appealing for help; and therefore they have spent money liberally in the endeavour to make St. George's as perfect as a hospital at the present day can be; and the paragraph of the report of the Prince's Fund which gives their reason for not making a grant shows that in the estimation of competent judges that endeavour has not been unsuccessful. But no riches can hold out against a constant excess of expenditure over income—for legacies are very uncertain, and the same error which prevents people from subscribing prevents them also from bequeathing.

"Much more might be said but for the fear of wearying the reader.

"One word in conclusion. It must be far from the mind of the managers of the Fund to injure a hospital of which the reigning Sovereign is always president, and of which His Royal Highness the Prince of Wales is a gracious and liberal supporter. Yet there can be no question that individual hospitals lose subscriptions in consequence of their friends preferring to give to the Fund, and some of our former subscribers have given this as their reason for ceasing to contribute. To the other hospitals this is more than recouped by the sums allotted from the Fund. But to St. George's, which receives nothing, the loss is uncompensated. The Governors, therefore, feel it their duty to do all in their power to urge on the public the need for increased support, and trust that they may have your powerful aid in that effort."

